# Age-adjusted *Plasmodium falciparum *antibody levels in school-aged children are a stable marker of microgeographical variations in exposure to *Plasmodium *infection

**DOI:** 10.1186/1471-2334-7-67

**Published:** 2007-06-29

**Authors:** Shona Wilson, Mark Booth, Frances M Jones, Joseph K Mwatha, Gachuhi Kimani, H Curtis Kariuki, Birgitte J Vennervald, John H Ouma, Eric Muchiri, David W Dunne

**Affiliations:** 1 Department of Pathology, University of Cambridge, Tennis Court Road, Cambridge, CB2 1QP, UK; 2 Kenya Medical Research Institute, Nairobi, Kenya; 3 Division of Vector Borne Diseases, Kenyan Ministry of Health, PO Box 54840, Nairobi, Kenya; 4 DBL – Institute for Health Research and Development, Jægersborg Alle 1D, 2920 Charlottenlund, Denmark; 5 Maseno University, Kisumu, Kenya

## Abstract

**Background:**

Amongst school-aged children living in malaria endemic areas, chronic morbidity and exacerbation of morbidity associated with other infections are often not coincident with the presence or levels of *Plasmodium *parasitaemia, but may result from long-term exposure to the parasite. Studies of hepatosplenomegaly associated with *Schistosoma mansoni *infection and exposure to *Plasmodium *infection indicate that differences that occur over 1–2 km in levels of *Plasmodium *transmission are related to the degree of exacerbation of hepatosplenomegaly and that *Plasmodium falciparum *schizont antigen (Pfs)-IgG3 levels may be a marker for the differing levels of exposure.

**Methods:**

To investigate the validity of Pfs-IgG3 measurements as a tool to assess these comparative exposure levels on a microgeographical scale, cross-sectional community surveys were conducted over a 10 × 6 km study site in Makueni District, Kenya, during low and high malaria transmission seasons. During both high and low malaria transmission seasons, thick blood smears were examined microscopically and circulating Pfs-IgG3 levels measured from dried blood spot elute. GIS techniques were used to map prevalence of parasitaemia and Pfs-IgG3 levels.

**Results:**

Microgeographical variations in prevalence of parasitaemia were observed during the high but not the low transmission season. Pfs-IgG3 levels were stable between high and low transmission seasons, but increased with age throughout childhood before reaching a plateau in adults. Adjusting Pfs-IgG3 levels of school-aged children for age prior to mapping resulted in spatial patterns that reflected the microgeographical variations observed for high season prevalence of parasitaemia, however, Pfs-IgG3 levels of adults did not. The distances over which age-adjusted Pfs-IgG3 of school-aged children fluctuated were comparable with those distances over which chronic morbidity has previous been shown to vary.

**Conclusion:**

Age-adjusted Pfs-IgG3 levels of school-aged children are stable and when mapped can provide a tool sensitive enough to detect microgeographical variations in malaria exposure, that would be useful for studying the aetiology of morbidities associated with long-term exposure and co-infections.

## Background

School-aged children in areas of stable malaria transmission are often immune to the severe complications attributable to the infection such as cerebral malaria and severe anaemia, as immunity to severe malaria develops after as few as one or two previous attacks [1]. School-aged children can nonetheless carry a burden of infection, not related to severe morbidity, as immunity to mild malaria and parasitaemia develops much more slowly [2] and on-going, long-term, exposure to *Plasmodium *infection can be responsible for, or along with co-infections, can contribute to, the development of more subtle morbidities such as chronic hepatosplenomegaly and mild/moderate anaemia. These more subtle morbidities are often not directly correlated with the presence or levels of parasitaemia [3-5].

Chronic hepatosplenomegaly, with the enlarged organs having a firm consistency, has been widely reported amongst school-aged children in *Plasmodium falciparum *endemic areas [3,6]. *Schistosomiasis mansoni *is also associated with childhood hepatosplenomegaly, in an intensity dependent manner. However, it has a higher prevalence in malaria endemic areas [7], and has been found to be associated with higher serum levels of *P. falciparum *schizont antigen (Pfs)-IgG3 [8]. Although Pfs-IgG3 is cross-reactive with *S. mansoni *adult worm antigen (SWA), its production is driven by *P. falciparum *infection rather than *S. mansoni *infection [9,10]. In, Makueni District, Kenya, a meso-endemic, seasonal transmission area, Booth and colleagues (2004) showed that, amongst 80 school-aged children, dry season Pfs-IgG3 levels were highest in those who resided within one kilometre of the only major water source. These Pfs-IgG3 levels were also significantly correlated with exacerbation of splenomegaly, which itself was both more prevalent and significantly more severe within a kilometre of the water source [11]. As transmission of *P. falciparum *is known to vary on a microgeographical scale in relation to mosquito breeding sites, due to mosquito host-seeking behaviour [12-14], this microgeographical pattern of Pfs-IgG3 level could reflect short-range differences in these children's exposure to *P. falciparum *infection. However, the decline of Pfs-IgG3 levels with distance of residence from the river described by Booth and colleagues, could not be confirmed as exposure-related, as neither parasitological nor entomological data were available.

Here we examine if finger-prick serum Pfs-IgG3 levels are (a) more temporally stable than blood smear detectable parasitaemia and (b) can be used, in areas with complex patterns of surface water distribution, to estimate relative exposure to *Plasmodium *infection on a microgeographical scale. Such characteristics would allow circulating Pfs-IgG3 to be used as a marker for assessing the contribution of chronic exposure to malaria towards chronic, subtle morbidities that do not necessarily coincide with current parasitaemia. To achieve this, community wide surveys were conducted in an area with a complex network of water bodies and Pfs-IgG3 levels were measured during both the low and high transmission seasons. Microgeographical fluctuations in Pfs-IgG3 were compared with spatial patterns of peripheral blood smear detectable parasitaemia at the same time points. Age-adjusted Pfs-IgG3 levels in children, but not adults, were found to be a more stable microgeographical marker of relative exposure than parasitaemia, pointing to the potential usefulness of the Pfs-IgG3 marker in studies of chronic, subtle morbidities that may be caused or exacerbated by *P. falciparum *and/or interactions with co-infecting pathogens.

## Methods

### Study site

**Figure 1:**
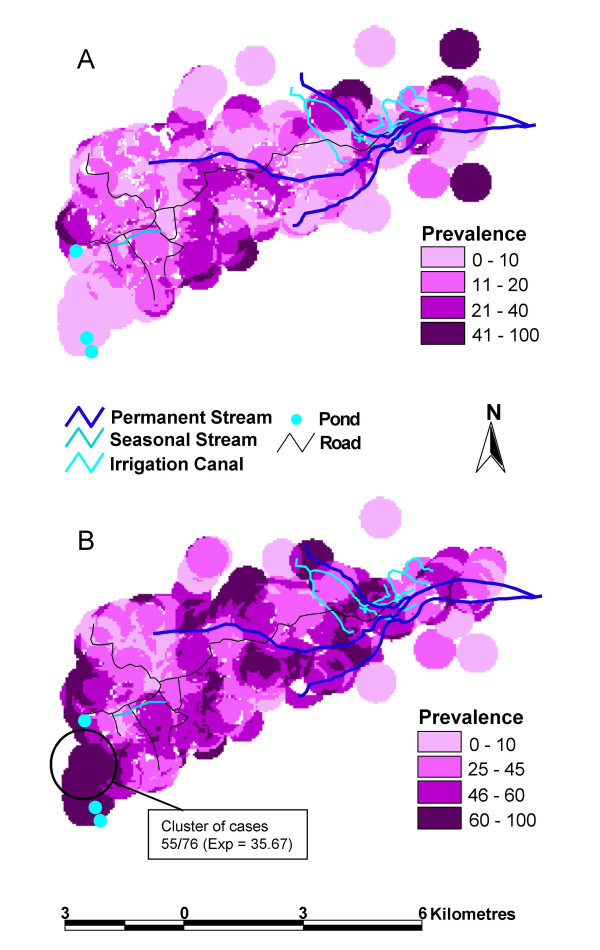
**Malaria prevalence maps for low and high transmission seasons**. Maps of malaria prevalence on a household level, as detected from thick blood smears, during (A) a low transmission season (n = 1044) and (B) a high transmission season (n = 973). Also shown is a cluster of households with a higher than expected prevalence of malaria during the high transmission season, detected using the Kulldorff scan technique (RR = 1.542, p = 0.004). Indicated for the cluster are the number of cases and the expected (exp) number of cases.

The study took place in the neighbouring Akamba communities of Yumbuni and Lower Mangelete, in Makueni District, Kenya; a rural area with small-scale agriculture. There were 493 households in the area (1 to 22 (mean = 4.31) occupants per household). Family groups often live within the same compound, and children attend one of 5 primary schools. The area is 800 m above sea level, with seasonal rains typically falling in November/December and April/May, though annual variations can occur, providing conditions suitable for stable but seasonal malaria transmission. *P. falciparum *is the most prevalent species, but *P. malariae *is also present. The transmitting mosquito vectors are *A. funestus *and *A. gambiae*, both are found to the east of the study area, while *A. gambiae *is the predominant vector in the west. Entomological inoculation rates (EIR) are not available for this area but it is considered to be meso-endemic. The study site was 10 km by 6 km, with the volcanic Chuylu Hills to the Northwest. Yumbuni, the community nearest to the Chuylu Hills is situated on lava flows that divert streams underground, so that the only surface water is a short stretch of a permanent stream, a seasonal stream and seasonal ponds, which dry up during the long dry season (fig. 1), resulting in arid conditions. In contrast, the lava flows do not extend to Lower Mangelete to the east, where the underlying geology allows a series of permanent streams to criss-cross the area (fig. 1). These, together with small irrigation canals, result in Lower Mangelete remaining green throughout the year.

All households in Yumbuni and Lower Mangelete were mapped using a Magellan 315 GPS. Signals from at least 5 satellites ensured a positional accuracy of within 5 metres. The stream courses and irrigational canals were mapped by taking co-ordinates at regular intervals (approx. every 100 m). Ponds and points of interest (joining of tributary, start of secondary canal etc) were recorded. Co-ordinates were downloaded using GPS Utility 4.04 (GPS Utility Ltd, Southampton, UK) and imported into ArcView (Version 3.2, ESRI, CA, USA) for visualisation and distance calculations. Distances were assigned to individuals using household demography data. Maps were generated using either the prevalence of parasitaemia or the mean antibody OD on a household basis. Households are mapped with a 0.5 km radius and when circles are overlapping, the average prevalence or mean antibody OD is shown.

### Study population

All adults who gave informed consent and all children, for whom their parents or guardians gave consent, were included in cross-sectional surveys. A thick blood smear for identification of *Plasmodium *infections was taken at each of two time points: November 2003, a low transmission time point and February 2004, a high transmission time point. The low transmission time point was at the end of a long dry period, which started in May 2003. The high transmission time point was after substantial rains in January 2004. A smear was considered negative if no *Plasmodium *parasite had been identified within 15 fields of view. Dried blood spots (DBS) were collected on filter papers (Schweitzer & Schell, Dassel, Germany) at both time points. 1044 individuals (age-range 3 to 94-yrs,) from 344 of the 493 households participated during the low transmission season survey, representing 34.0% of the total population. 973 individuals (age-range 3 to 95-yrs), from 369 of 493 households participated during the high transmission survey, representing 31.7% of the total population. 684 individuals, from 293 households, participated in both surveys. At both time points the sample population differed significantly from the total population in terms of age and sex, as adult males were under represented. There was good compliance amongst school-aged children (5 to 17-yrs), with over 60% of school-aged children participating at one or other time point, and 47.1% participating at both time points. *S. mansoni *infection intensities had previously been surveyed; participants provided two stool samples and 2 Kato Katz slides [15] were prepared from each. All individuals presenting with clinical malaria were treated, as were *S. mansoni *infections and minor ailments. The study received approval from the Kenya Medical Research Institute National Ethical Review Committee.

### Antigen preparations

The Pfs Ag was prepared as previously described [9]. Briefly, Pfs Ag was produced by harvesting mature schizonts from synchronised A4 strain *Plasmodium falciparum *cultures, by centrifugation at 2000 rpm through 60% Percoll (Sigma, Poole, Dorset, UK). The schizont layer was resuspended at a concentration of 1 × 10^8 ^schizonts/ml, aliquoted into cryotubes, freeze-thawed twice and stored at -80°C until use. *S. mansoni *worm Ag (SWA) was prepared as previously described [16].

### Dried blood spot elution and antibody ELISA

A pre-study trial comparing Ab levels measured from plasma samples and whole blood spots dried onto filter papers showed that although there was a need for a reduced sample dilution factor with DBS elute, correlations between the two methods of collection were high (r = 0.899). Samples were allocated randomly for individual and survey into 96-well formats. Replicate European plasma samples were added to each plate to ensure standardisation of readings between plates, but were not sufficient in number to define a positive cut-off. One 6 mm diameter disc was punched out from a DBS for each individual study participant and eluted in 100 μl PBS/0.3% TNBP/1% Tween 80/0.02% sodium azide (all Sigma). Samples were shaken at room temperature for 5 min and incubated overnight at 4°C. Supernatants were stored at -80°C until use.

ELISA were carried out in duplicate. 50 μl of 8 μg/ml Pfs Ag, or 50 μl of 10 μg/ml SWA, in bicarbonate coating buffer were coated onto Immulon 2 plates (Thermo Labsystems, Franklin, MA, USA) and incubated overnight at 4°C. Plates were blocked with 1% milk powder (Marvel, Spalding, Lincs, UK). 50 μl of DBS elute diluted 1:10 for IgG3 assays and 1:25 for IgG4 assays was incubated overnight at 4°C. Antigen specific antibodies were detected using biotinylated anti-human IgG3 (Zymend, San Francisco) at a 1:500 dilution, or biotinylated anti-human IgG4 (BD Pharmingen, San Diego) at a 1:2000 dilution, followed by 1:3000 poly-HRP (CBA, Amsterdam, Netherlands). Assays were developed with 2× *o*-phenylenediamine (Sigma) and read at a dual wavelength of 360 nm and 490 nm. Specific antibody levels were expressed as the mean OD of duplicate assays.

### Statistical analysis

Statistical analysis was carried out using SPSS 12 for Windows (SPSS Inc., Chicago, USA). Participants were divided into three-age groups (<5-yrs, 5- to 17-yrs and >17-yrs). Chi-squared analysis was used to determine differences in malaria prevalence by age and by distance of residence from water bodies. Paired students t-tests were used to analyse longitudinal differences in Pfs-IgG3 levels. Differences in Pfs-IgG3 of individuals by age and by distance of residence from water bodies were analysed by ANOVA with Hochberg GT2 post-hoc analysis, as suitable for groups containing different sample numbers. Pfs-IgG3 levels of individuals with and without detectable parasitaemia were compared by Students t-test. Correlations between Pfs and SWA antibody levels were calculated using Spearman's Rank correlations. Pfs-IgG3 was log_10 _transformed and adjusted for age using split linear regression prior to mapping. Loess lines were fitted to scatter plots of age against transformed Pfs-IgG3 to determine at which age to split the linear regression (<= 17-yrs and >17-yrs). Analysis of spatial clustering used SaTScan™ software [17]. Most likely clusters of cases are detected using a circular window that scans the study area systematically, and significant increases in prevalence are detected by calculation of likelihood ratio for each window. The pre-determined upper-limit for the size of the window was set at 25% and it was specified that clusters should not overlap geographically. The Bernoulli model was used, as it is appropriate for binomial data.

## Results

### Prevalence of *P. falciparum *infections

The overall prevalence of peripheral blood smear detectable parasitaemia was 14.0% at the low transmission season time-point and 47.7% at the high transmission time-point. During the low transmission season a significantly greater proportion of <5-yr olds and 5- to 17-yr olds had detectable *P. falciparum *infections than adults (χ^2 ^= 9.668, p = 0.002 and χ^2 ^= 6.430, p = 0.011 respectively). The prevalence of parasitaemia was 26.5% in the <5-yr olds, 15.3% in school-aged children (5- to 17-yrs), and 11.3% in adults. During the high transmission season the prevalence of parasitaemia was 42.5% in the <5-year olds, 51.8% in school-aged children, and 42.1% in adults. The prevalence of parasitaemia was not significantly different between <5-yr olds and adults (χ^2 ^= 0.210, p = 0.646) during the high transmission season, but remained significant between school-aged children and adults (χ^2 ^= 8.523, p = 0.004).

Low and high transmission season maps of *P. falciparum *prevalence were drawn using the prevalence per household (Fig. 1). During the low season, there was little variation in malaria prevalence over the study area (Fig. 1a). The Kulldorff scan technique was used to detect household clusters that had significantly higher *P. falciparum *prevalence amongst their inhabitants than would be expected by chance. This confirmed that no part of the area had a significantly higher prevalence of blood smear detectable malaria during the low transmission season (most likely cluster; RR = 3.897, p = 0.087). However, spatial trends in transmission were present in the high transmission season (Fig. 1b). Prevalence of parasitaemia was >45% around the permanent streams. Within the western area a prevalence above >45% was only observed around the seasonal stream and between two season ponds located to the west and southwest, where it was >60%. The Kulldorff scan technique detected a household cluster in the southwest of the area, between these ponds, which had a significantly higher prevalence of parasitaemia (RR = 1.542, p = 0.004).

**Figure 2:**
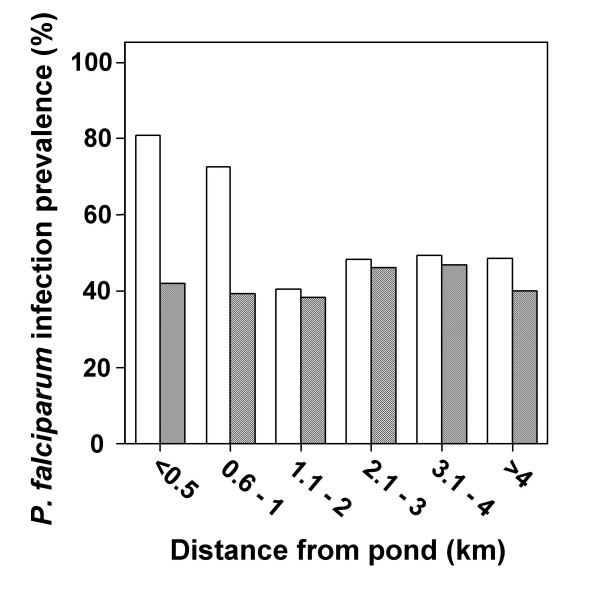
**High transmission season prevalence of parasitaemia with increasing distance from the nearest pond**. Prevalence of *Plasmodium falciparum *parasitaemia is shown for school-aged children (5- to17-yrs, n = 560; clear bars) and adults (>17-yrs, n = 380; striped bars). Significant differences in prevalence were determined by Chi-squared analysis. *Significantly higher prevalence of malaria infection than further from the ponds (p < 0.001).

In concurrence with the cluster analysis, there was no significant relationship between the distance of an individuals' residence from the nearest pond and prevalence of parasitaemia, during the low transmission season (χ^2 ^= 1.680, p = 0.891). However, prevalence during the high transmission season was significantly greater for individuals living within 1 km of the nearest pond (χ^2 ^= 16.460, p = 0.006). Although there was a significant difference in prevalence of parasitaemia, with distance from the nearest permanent stream, during the high transmission season (χ^2 ^= 20.559, p < 0.001), post-hoc analysis indicated that this was due to the higher prevalence amongst individuals who resided 3.5–4 km from the streams. Those who resided 3.5–4.5 km from a permanent stream were the same individuals who lived within a kilometre of a pond. As prevalence of parasitaemia was age-related during the high transmission season, individuals were grouped according to whether they were school-aged (5- to 17-yrs) or adults (>17-yrs). There were insufficient <5-years olds to include them in this further analysis (n = 33 for the high transmission season). The relationship between distance of residence from nearest pond and prevalence of parasitaemia during the high transmission season (fig. 2) remained significant for school-aged children (χ^2 ^= 27.787, p < 0.001), but not for adults (χ^2 ^= 2.119, p = 0.832). When analysis was repeated solely on individuals who participated in both surveys, results were similar (data not shown).

### Pfs-IgG3 levels

**Figure 3:**
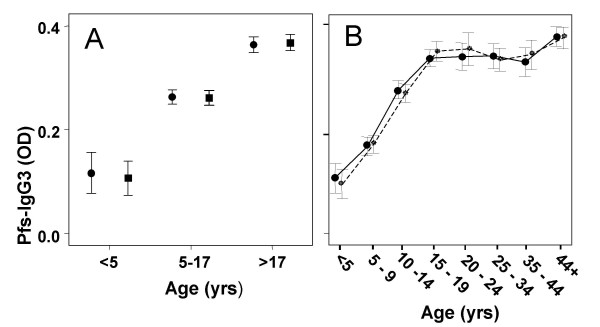
**Age profiles of Pfs-IgG3 levels during low and high transmission seasons**. (A) Longitudinal comparisons of mean +/- 2 standard errors low transmission season (circles) and high transmission season (squares) *Plasmodium falciparum *schizont Ag (Pfs)-IgG3 levels of individuals who participated at both time points (n = 684), stratified by age. Paired t-tests found no significance between low and high malaria transmission season Pfs-IgG3 levels for any age group. (B) Cross-sectional age-related curves of low transmission (circles, solid line; n = 1044) and high transmission (squares, dashed line; n = 973) Pfs-IgG3 levels; bars represent +/- 2 standard errors.

**Figure 4:**
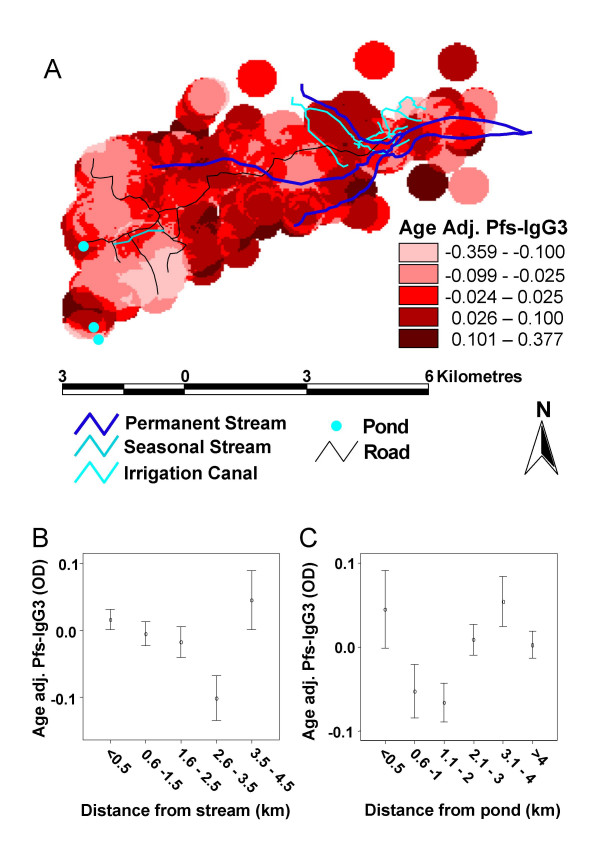
**Microgeographical patterns in age adjusted Pfs-IgG3 levels**. (A) Map of mean age-adjusted low transmission season *Plasmodium falciparum *schizont antigen (Pfs)-IgG3 levels (n = 1044), on a household level. Pfs-IgG3 levels were age-adjusted by linear regression which was split at <= 17-yrs and >17-yrs. (B) Age-adjusted Pfs-IgG3 levels with distance of residence from the nearest permanent stream. Shown are the mean levels +/- 2 standard errors. (C) Age-adjusted Pfs-IgG3 levels with distance of residence from the nearest seasonal pond. Shown are the mean levels +/- 2 standard errors.

There was no significant difference between high and low season Pfs-IgG3 levels for any age group (Fig. 3a). However, children's Pfs-IgG3 levels increased with age, in both high and low malaria transmission seasons, reaching a plateau in late adolescence/early-adulthood (Fig. 3b). These age-related variations in Pfs-IgG3 levels were significant during both the low and high transmission seasons (F = 105.225, p < 0.001 and F = 114.863, p < 0.001 respectively). Pfs-IgG3 levels were not significantly associated with detectable parasitaemia (low season, t = -0.728, p = 0.467; high season, t = -0.666, p = 0.510). As Pfs-IgG3 levels did not fluctuate between seasons, low transmission season levels were mapped to determine if accumulative spatial differences in malaria exposure could be observed. Low-season Pfs-IgG3 levels were adjusted for age prior to mapping, as the strong Pfs-IgG3 association with age could lead to underlying differences in the age structure within the study area confounding any spatial relationship with exposure. Pfs-IgG3 levels decreased in a southwesterly direction, as distance from the permanent streams increased, but levels rose again in the far south west of the study area (Fig. 4a). Mean individual age-adjusted Pfs-IgG3 levels decreased with distance from the permanent streams (Fig. 4b) reaching their lowest levels 2.5–3.5 km from the streams, before increasing again 3.5–4.5 km from the nearest permanent stream. The distance from the streams at which Pfs-IgG3 levels increased was close to the ponds, as clarified by the stratification of age-adjusted Pfs-IgG3 levels with distance from nearest pond (Fig. 4c). Pfs-IgG3 was high within 0.5 km of the ponds, but decreased sharply before increasing again at the distance equivalent to the location of the permanent streams.

**Figure 5:**
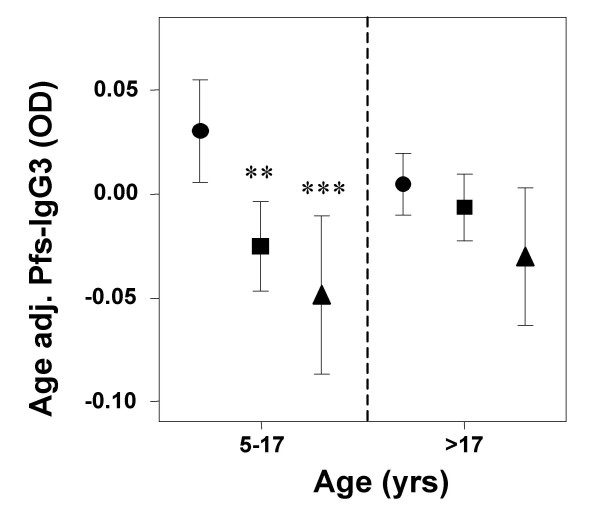
**Microgeographical patterns in age adjusted Pfs-IgG3 levels stratified by age**. Shown are the mean +/- 2 standard errors age adjusted low transmission season *Plasmodium falciparum *schizont antigen (Pfs)-IgG3 levels of school-aged children (5–17 yrs, n = 574) and adults (>17 yrs, n = 435), who live <0.5 km, 0.6–1.5 km and >1.5 km from the nearest water body (either a permanent stream or a seasonal pond). Statistical significance was determined by ANOVA with Hochberg GT2 post-hoc analysis. **Indicates a significantly lower mean age-adjusted Pfs-IgG3 level than those who live within 0.5 km of the nearest water body, p < 0.01, ***p < 0.001.

As permanent streams and ponds influenced age-adjusted Pfs-IgG3 levels, distances from ponds to the households and distances from permanent streams to households, were combined to produce a new variable, 'distance from a water body'. Distances were stratified into <0.5 km, 0.6–1.5 km and >1.5 km from nearest water body. In school-aged children (5- to 17-yrs), age-adjusted Pfs-IgG3 levels decreased significantly as distance to nearest water body increased (Fig. 5). Age-adjusted Pfs-IgG3 levels of children living 0.6-1.5 km and >1.5 km from nearest water body were significantly less than those of children living within 0.5 km of nearest water body (F = 8.345, p < 0.001; Hochberg GT2 post hoc, p = 0.003 and p < 0.001 respectively). Adult (>17-yrs) Pfs-IgG3 levels did not significantly decrease with distance of residence from nearest water body (F = 2.190, p = 0.113). When analysis was repeated solely on individuals who participated in both surveys, results were similar (data not shown). Additionally, although, the mapped age-adjusted Pfs-IgG3 levels also appear raised close to the seasonal stream, addition of this into the variable "distance from water body" did not influence the analysis (data not shown).

### Correlations between Pfs- and schistosome-antibody levels

SWA-IgG3 and SWA-IgG4 levels were also measured. Pfs-IgG3 levels were highly correlated with SWA-IgG3 levels in this study population during the low and the high transmission seasons, but were weakly correlated with SWA-IgG4 levels (Table 1). SWA-IgG4 responses and *S. mansoni *faecal egg counts, when mapped, were highest over the eastern end of study area, around the irrigation canals and network of permanent streams (data not shown), the only part of the study area where *Biophalaria *intermediate hosts of *S. mansoni *were found (authors' unpublished results). This area did not correspond with areas of higher levels of age-adjusted Pfs-IgG3, indicating that the presence of *S. mansoni *did not influence Pfs-IgG3 levels.

**Table 1:** Correlations between Pfs-IgG3 levels and SWA-IgG3 and IgG4 levels

	**Transmission Season**	
**Antibody**	**Low**	**High**
SWA-IgG3	0.694	0.680
SWA-IgG4	0.203	0.245

Shown are the Spearman's Rank correlation co-efficients for the associations between *Plasmodium falciparum *schizont Ag (Pfs)-IgG3 levels and *Schistosoma mansoni *adult worm Ag (SWA) specific IgG3 and IgG4 levels during low and high malaria transmission time points. All correlations were significant (p < 0.001).

## Discussion

The aim of the present study was to identify a serological marker of accumulative exposure to *Plasmodium *infections, for use in studies of subtle morbidities, such as chronic hepatosplenomegaly, that are often not associated with the presence of peripheral blood detectable parasitaemia, but vary over a microgeographical scale. It was demonstrated that age-group mean Pfs-IgG3 levels were stable between low and high transmission seasons, even though the prevalence of blood smear detectable parasitaemia varied significantly. Low transmission season Pfs-IgG3 was measured in finger-prick samples taken at the end of the dry season, when prevalence of parasitaemia confirmed transmission across the area was low. This indicates that Pfs-IgG3 levels are a more stable marker of exposure than prevalence of parasitaemia. Therefore, even though on a community level, maximum prevalence of detectable malaria parasitaemia is strongly associated with annual EIR [18], the serological marker is preferable to measuring peak parasitaemia; particularly when considering the caveats of (a) the highly seasonal nature of peak prevalence of detectable malaria in many endemic areas, (b) the reflection of short term, rather than long-term, transmission patterns in parasitaemia prevalence in young children [19] and (c) the potential confounding by the development of immunity, as shown by children living closest to mosquito breeding sites, in areas of high transmission, having a lower prevalence of parasitaemia [13].

Pfs-IgG3 levels reached a plateau in >17-yr olds, suggesting that the response reaches saturation in adults. This observed saturation of the IgG3 response in >17-yr olds resulted in a loss of statistical significance, in the relationship between age-adjusted Pfs-IgG3 and distance of residence from nearest water body. A spatial trend of decreasing prevalence of parasitaemia with distance of residence from nearest seasonal pond during the high transmission season was also not significant for adults. This, along with the significantly lower prevalence of parasitaemia in adults, during both low and high transmission seasons, indicates that spatial patterns in exposure cannot be serologically determined, using the present technique, from surveys of immune adults. Response saturation has previously been reported as a caveat to serological markers of exposure to malaria over macrogeographical distances [20]. This is particularly true if using sero-conversion rates, which are defined by detection of a response rather than the magnitude of the response.

For school-aged children, in whom antibody levels were still increasing, age-adjusted Pfs-IgG3 levels declined sharply with distance from potential mosquito breeding sites. The distances over which a significant decrease is observed is comparable, particularly in relation to the seasonal streams in the west where *A. gambiae *is the predominant vector, with the distances that entomological differences have been observed, as a capture study in Western Kenya showed that 90% of adult *A. gambiae *mosquitoes are found in households located within 300 m of the nearest larval breeding site [21], and studies elsewhere in Africa have confirmed that mosquito dispersal is less than 1 km [22,23]. Mapping school-aged children's serological responses is, therefore, likely to be an approach that is applicable to studies in other endemic areas, where internal comparisons of relative exposure to *Plasmodium *infections would be informative.

Assessment of serological markers of exposure in children <5-yrs would also be beneficial, particularly as it is known that within this age group, levels of transmission can have a great impact on the incidence and type of severe complications that occur due to malaria [24]. Whether or not this technique would be applicable for children less than 5-yrs of age, the age group who suffer the severest consequences of *Plasmodium *infections, could not be determined from the present study, due to small number of children in this age group who participated. However, it is known that IgG3 responses to malaria antigens are the slowest to develop, with predominance of IgG3 over IgG1 to MSP-2, an antigen that preferentially induces IgG3 responses, not being fully established until late adolescence, and IgG1 being the predominant response in very young children (<2 yrs-age) [25,26]. In young children IgG1 responses may therefore be better marker of exposure. However, in the present study Pfs-IgG1 levels, measured but not reported here, had similar results in relationship to spatial patterns of exposure, with age-adjusted levels for school-aged children but not adults being significantly associated with distance of residence from the nearest water body.

Finally, even though cross-reactivity between *Plasmodium *and *S. mansoni *infections is known to occur [9,10], SWA-IgG4 levels, which have been shown to correlate with schistosome infection intensities in many settings [27-32], were only weakly correlated with the Pfs-IgG3 levels and were raised in a different part of the study area to Pfs-IgG3, indicating that the approach was not influenced by the presence of schistosomiasis.

## Conclusion

The present study shows that Pfs-IgG3 levels can be used during both the low and high transmission seasons to detect variation in exposure, particularly if population means are used, as levels may remain stable on a population level while fluctuating in individuals; and, important for use in studies of morbidity related to chronic exposure, Pfs-IgG3 levels are more temporally stable than prevalence of parasitaemia. Age-adjusted Pfs-IgG3 levels of school children were however, sensitive to fluctuations in exposure over short distances; distances that were comparable with those over which chronic morbidity varies [11] and detectable even though there was a relatively complex distribution of potential mosquito breeding sites within the study area. The approach was also found not to be influenced by the presence of schistosomiasis. It is, therefore, ideal for use in microgeographical-epidemiological studies of exposure-related phenomena, that are dependent on continuing exposure to malaria, such as the development of hepatosplenomegaly and interactions with co-exposure to other pathogens, like *Schistosoma *species, that have their own complex spatial patterns of transmission.

## Competing interests

The author(s) declare that they have no competing interests.

## Authors' contributions

SW participated in field activities, undertook the serology, analysis and drafted the manuscript. MB participated in the design of the study and participated in fieldwork. FMJ prepared the antigens and participated in the serology. JKM, GK, HCK, JHO and EM participated in the planning and execution of field activities. BJV and DWD participated in the design of the study and critically revised the manuscript. All authors have read and approved the manuscript.

## Pre-publication history

The pre-publication history for this paper can be accessed here:


